# Artesunate, an Anti-Malaria Agent, Attenuates Experimental Osteoarthritis by Inhibiting Bone Resorption and CD31^hi^Emcn^hi^ Vessel Formation in Subchondral Bone

**DOI:** 10.3389/fphar.2019.00685

**Published:** 2019-06-14

**Authors:** Yicheng Li, Wenbo Mu, Boyong Xu, Jiangdong Ren, Tuerhongjiang Wahafu, Shalitanati Wuermanbieke, Hairong Ma, Hongwei Gao, Yang Liu, Keyuan Zhang, Abdusami Amat, Li Cao

**Affiliations:** ^1^Department of Orthopaedics, First Affiliated Hospital of Xinjiang Medical University, Urumqi, China; ^2^State Key Laboratory of Pathogenesis, Prevention and Treatment of High Incidence Diseases in Central Asian Xinjiang Key Laboratory of Echinococcosis, Clinical Medical Research Institute, First Affiliated Hospital of Xinjiang Medical University, Urumqi, China; ^3^School of Life Sciences, Ludong University, Jinan, China

**Keywords:** artesunate, bone resorption, CD31^hi^Emcn^hi^ vessel formation, subchondral bone, osteoarthritis

## Abstract

Osteoarthritis (OA) is a common and debilitating joint disease worldwide without interventions available to reverse its progression. Artesunate (ART), an anti-malaria agent, possesses diverse biological activities, including the inhibition of osteoclastogenesis and angiogenesis in various cells, but its role in subchondral bone during OA progression is not known. Here, we explored the curative effects of ART on the pathogenesis of OA in anterior cruciate ligament transection (ACLT) mice models. We found that ART attenuated articular cartilage degeneration, defined by lowered histologic scoring of OA and retarded calcification of the cartilage zone. Moreover, ART improved the expression of lubricin and aggrecan and reduced the expression of collagen X (Col X) and matrix metalloproteinase-13 (MMP-13). In parallel, ART normalized abnormal subchondral bone remodeling by maintaining bone volume fraction (BV/TV) and subchondral bone plate thickness (SBP Th) and reducing trabecular pattern factor (Tb.pf) compared to the vehicle-treated mice. Our results indicated that ART suppressed osteoclastic bone resorption through regulating RANKL-OPG system, restored coupled bone remodeling by indirectly inhibiting TGF-β/Smad2/3 signaling. Additionally, ART abrogated CD31^hi^Emcn^hi^ vessel formation *via* downregulating the expression of vascular endothelial growth factor (VEGF) and angiogenin-1 in subchondral bone. In conclusion, ART attenuates ACLT-induced OA by blocking bone resorption and CD31^hi^Emcn^hi^ vessel formation in subchondral bone, indicating that this may be a new therapeutic alternative for OA.

## Introduction

Osteoarthritis (OA) is a common multifactorial degenerative joint disease, which will affect 67 million people in the United States by 2030 (Hootman and Helmick, 2006). It is pathologically characterized by inflammatory synovium, articular cartilage degeneration, osteophyte formation, and subchondral bone edema and sclerosis (Hutton, 1989), all of which result in chronic arthralgia, stiffness, and irreversible arthrosis dysfunction (Goldring and Berenbaum, 2004). Data from the Global Burden of Disease 2010 study showed that OA of hip and knee was ranked as the 11th highest contributor to global disability and 38th highest in disability-adjusted life years (Cross et al., 2014). Although a range of interventions for treating OA have been employed, including physical activity (Fernandopulle et al., 2017), extracorporeal shock wave (Imamura et al., 2017), and analgesics and nonsteroidal anti-inflammatory drugs (NSAIDs) (Le Graverand-Gastineau, 2010), these treatments only temporarily relieve clinical symptoms but fail to decelerate or reverse disease progression. Therefore, there is an urgent need for more preventive and disease-modifying therapies.

OA was initially seen as a major disease of articular cartilage, but the role of subchondral bone on OA initiation and progression is of great interest (Li et al., 2013; Muratovic et al., 2018). Subchondral bone is in the dynamic remodeling to guarantee stabilization of the subchondral bone microstructure (Goldring, 2012). In this process, the activity of osteoclasts and osteoblasts are temporally and spatially regulated, thus coupling bone remodeling (Madry et al., 2010). However, osteoclastic bone resorption dramatically increases following abnormal mechanical stress, resulting in the release of excessive active transforming growth factor-β (TGF-β) from the bone matrix (Tang et al., 2009). Emerging evidence demonstrates that abnormally activated TGF-β1 elicit uncontrolled recruitment of mesenchymal stem cells (MSCs) and formation of osteoid islets (Cui et al., 2016), which not only uncouples bone formation and resorption but also contributes to the subchondral osteosclerosis in OA animal models (Cao, 2011). Additionally, CD31^hi^Emcn^hi^ vascular congestion in subchondral bone has also been noted in OA (Cui et al., 2016), which indicates its capacity to coupling osteanagenesis (Kusumbe et al., 2014). Moreover, various inflammatory mediators released from aberrant bone remodeling areas reach overlying cartilage *via* capillaries to exacerbate inflammatory responses (Lajeunesse and Reboul, 2003). Therefore, it is essential to carry out preemptive therapy aimed at the multiple pathomechanism in subchondral bone.

Artemisinins derived from *Artemisia annua* have been used to treat malaria in traditional Chinese herb (Jiang et al., 2018). Artesunate (ART), a semisynthetic derivative of artemisinin, is the most investigated agent due to its water solubility and high bioavailability in the treatment of malaria (Newton et al., 2000). In recent years, ART has received more attention due to its pharmacological actions beyond being an anti-malarial drug. ART has been reported to suppress the receptor activator of nuclear factor-κB ligand (RANKL)-induced osteoclastogenesis, the expression of osteoclastic-specific genes and the resorption pit formation, as evidenced by rat models of osteolysis and osteoporosis (Zeng et al., 2017; Wei et al., 2018). Specifically, ART effectively regulates the serum RANKL-osteoprotegerin (OPG) system by reducing RANKL level and restoring OPG level (Zeng et al., 2017). Furthermore, ART is effective in regulating the TGF-β/Smads signaling to resist lung fibrosis (Li et al., 2014; Wang et al., 2015), which is critical for the migration and differentiation of MSCs in subchondral bone. In addition, ART exerts anti-angiogenic effects *via* the inhibition of vascular endothelial growth factor (VEGF) (Verma et al., 2017) and the downregulation of placental growth factor inducing osteogenic differentiation of mesenchymal progenitors during bone remodeling (De, 2012; Vandewynckel et al., 2014). As described above, ART seemingly shows the potential effects for targeting the multiple pathological changes of OA. Although the ART-mediated amelioration of OA pathogenesis has been reported (Zhao et al., 2017), its effects on subchondral bone remains unclear.

In this study, we aimed to investigate whether ART attenuates OA progression, including ameliorating articular cartilage degeneration and subchondral bone sclerosis, in mice anterior cruciate ligament transection (ACLT) models by blocking bone resorption, Smad2/3 phosphorylation, and CD31^hi^Emcn^hi^ vessel formation.

## Materials and Methods

### Animals

All procedures were carried out in accordance with the guidelines of the Association for Assessment and Accreditation of Laboratory Animal Care, and the experiment was approved by the Institutional Animal Care and Use Committee of the First Affiliated Hospital of Xinjiang Medical University (protocol number IACUC20171129-01). C57BL/6J mice (3-month-old, male) were purchased from Vital River (Beijing, China) and given 7 days of feeding and housing adaptation. Environmental conditions include the following: temperature 25 ± 2°C, humidity 55 ± 5%, light/dark cycle 12 h, all mice free to chow and water *ad libitum*. A preliminary experiment was performed first to identify the optimal dose. The mice were randomly assigned to the sham group, vehicle group, and multiple concentrations of ART group (25, 50, 100, and 200 mg/kg; n = 10 per group). Then, the mice received an ACLT of the right knee to establish the OA model. In the sham group, the right joint capsule was opened, the ACL was exposed, and the incision was sutured. Beginning on the second day after ACLT operation, ART (Wanxiang Hengyuan Technology Co., Ltd., Tianjin, China) or equivalent volume of vehicle (5% NaHCO_3_) was treated daily by intraperitoneal injection for 60 days. In the formal experiment, all mice were assigned to the sham group, vehicle group, and optimal dose of ART group (n = 50 per group) randomly. Ten mice were euthanized at 0, 14, 30, and 60 days postoperation; another 10 mice in each group were euthanized for angiography at postoperative day 30.

### Histological Analysis and Immunostaining

Following euthanasia, the right knee joints of mice were excised and fixed in 10% buffered formalin for 24 h. After that, the knee joints were decalcified with 10% EDTA (pH 7.3) for 3 weeks and then embedded in paraffin. Sections (4 μm) of the knee joint medial compartment were processed for hematoxylin and eosin (HE), safranin O staining, and immunostaining. The thickness of the hyaline cartilage (HC) and calcified cartilage (CC) were measured with HE staining. Tartrate-resistant acid phosphatase (TRAP) staining was performed following a manufacturer protocol (Sigma-Aldrich).

The staining method was described previously (Wei et al., 2017). Briefly, sagittal sections of the knee joint medial compartment were rehydrated and endogenous peroxidase was quenched with 3% H_2_O_2_, followed by treatment with 0.1% trypsin for 30 min at 37°C for antigen retrieval. To reduce nonspecific staining, sections were blocked at 37°C with 20% normal horse serum diluted in 3% BSA for 30 min. Then, sections were incubated with primary antibodies against lubricin (Abcam, 1:200, ab28484), aggrecan (Abcam, 1:100, ab216965), collagen X (COL X, Abcam, 1:100, ab58632), matrix metalloproteinases-13 (MMP-13, Abcam, 1:100, ab39012), osterix (Abcam, 1:400, ab22552), TGF-beta 1 (Abcam, 1:40, ab92486), phosphorylated Smad2/3 (pSmad2/3, Santa Cruz Biotechnology Inc., 1:40), CD31 (Abcam, 1:20, ab28364), endomucin (Emcn, Santa Cruz Biotechnology Inc., 1:100, sc-53940), VEGFA (Abcam, 1:100, ab9570), and angiopoietin-1 (Ang-1, Abcam, 1:100, ab102015) at 4°C overnight. For immunohistochemical staining, a horseradish peroxidase streptavidin detection system (ZSGB BIO) was used to detect the immunoactivity, followed by counterstaining with hematoxylin (ZSGB BIO). For immunofluorescent staining, secondary antibodies conjugated with fluorescence were incubated for 1 h at room temperature (RT) while avoiding light. Histomorphometric measurement was performed on the medial tibial subchondral bone (Olympus DP26), and quantitative analysis was conducted in a blinded way with cellSens software (Olympus, Int). The number of positively stained cells in the subchondral bone or cartilage area was counted from five sequential sections per specimen in each group. The histologic scoring of OA in the medial tibial plateau was calculated as described by Glasson et al. (2010).

### Serum Measurements

TRACP5b and cathepsin K levels in blood serum are able to reflect osteoclast activity. These factors were detected using an enzyme-linked immunosorbent assay (ELISA) kit according to the instructions from the manufacturer (CUSABIO, China). ELISA results were quantitated by absorbance at 450 nm on a microplate reader (Bio-Rad, Hercules, CA, USA) and normalized by the number of cells per well (Kihara et al., 2017).

### Molecular Modeling

Molecular docking was executed for accurate docking of the ligand into the protein active sites using the LibDock module in Discovery Studio (Dassault Systèmes BIOVIA, Discovery Studio Modeling Environment, Release 2017, San Diego: Dassault Systèmes, 2016). The 3D crystal structures of mice RANK and TGF-β receptor I domains were obtained from the Protein Data Bank (RANK PDB ID: 1LB5, TGF-β receptor I domains PDB ID: 5E8S). The protein was prepared by removing all water compounds and processed by the protocol of Prepare Protein. The CHARMM force field was applied using the receptor–ligand interactions tool in Discovery Studio Visualizer 2.0. All default parameters were used during the docking after protein preparation.

### Micro-CT Analysis

As described in previous studies (Mu et al., 2018), the knee joints without excess soft tissue of mice were preserved in 10% buffered formalin overnight and were analyzed by a high-resolution micro-CT (µCT) (SkyScan 1172). The data were subsequently reconstructed (NRecon v1.6), analyzed (CTAN, v1.9), and reestablished for 3D model visualization (CTVol, v2.0). The sagittal view of the entire medial compartment of the tibial subchondral bone was selected for 3D histomorphometric analysis. The whole tibial subchondral bone medial compartment was identified as a region of interest. Three-dimensional structural parameters, including bone volume fraction (BV/TV), trabecular pattern factor (Tb.pf), and subchondral bone plate thickness (SBP Th.), were analyzed.

### Micro-CT-Based Microangiography

The mice were euthanized, the thoracic cavity was opened, and the right atrium was punctured. The vascular circulation system was flushed with 0.9% normal saline solution containing heparin sodium (100 U ml^-1^) through a needle that was inserted into the left ventricle. Then, the vascular circulation system was flushed with 10% neutral buffered formalin through the same needle, followed by the radiopaque silicone rubber compound containing lead chromate (MICROFIL MV-122, Flow Tech). The specimens were stored at 4°C overnight, and then the knee joints were collected and fixed in 10% neutral buffered formalin for 4 days. The specimens were decalcified in a rapid acid decalcifier solution (Rapid Cal Immuno, ZSGB-BIO) for 2 days to facilitate image threshold of the vasculature from the surrounding tissues. Images were obtained from a high-resolution micro-CT imaging system (SkyScan 1172) with a resolution of 9-μm isotropic voxel size (Ji et al., 2018).

### Statistical Analysis

The results are presented as means ± standard deviation (SD). One-way ANOVA was preformed to analyze the statistical difference among different groups. The level of significance was set at *p* < 0.05. All data analysis was carried out using SPSS 18.0 analysis software (SPSS Inc., Chicago, Illinois).

## Results

### ART Alleviates the Degradation of Articular Cartilage in ACLT Mice

To study the chondroprotective effects of ART, we administered ART intraperitoneally in ACLT mice. First, we designed multiple doses of ART to identify the optimal dose in preliminary experiment. HE staining showed that the thickness of CC was increased in the vehicle and lower concentration groups (25 or 50 mg/kg) and maintained a similar level between the sham and higher concentration groups (100 or 200 mg/kg) (**Supplementary Figure 1A, C**). In parallel with histologic scoring of OA, safranin O staining indicated that lower concentration groups had minimal chondroprotective effects and greater concentration (200 mg/kg) negatively affected proteoglycan production in articular cartilage (**Supplementary Figure 1B, D**). In addition, the liver coefficient was evaluated in each dose of mice, showing that no significant differences were observed among the three groups (25, 50, and 100 mg/kg) (**Supplementary Figure 1E**). However, the mice administered 200 mg/kg exhibited lower liver coefficients than the other groups (**Supplementary Figure 1E**). Therefore, the optimal dose was 100 mg ART per kg body weight injected daily.

**Figure 1:**
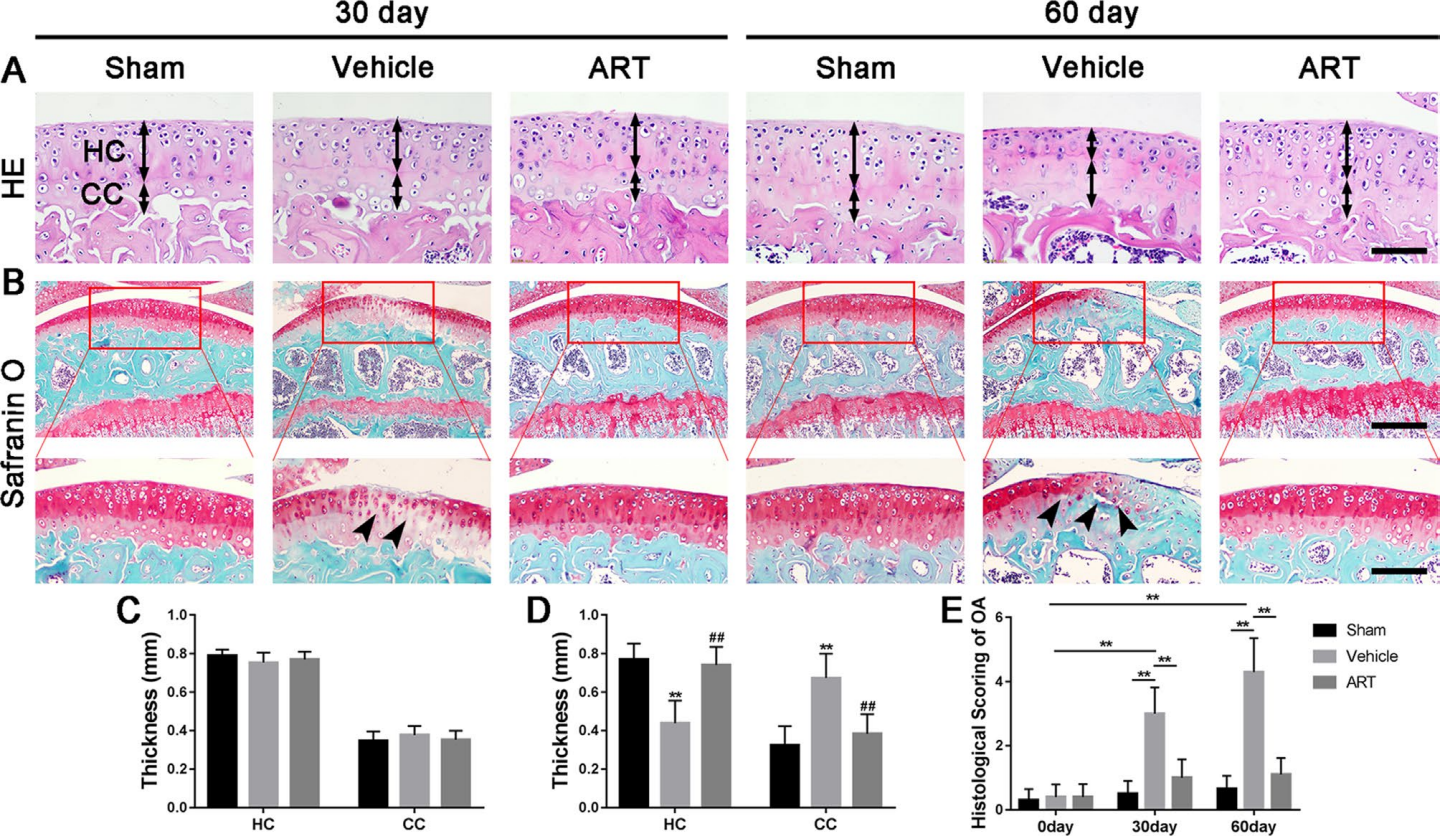
Artesunate (ART) attenuates articular cartilage degeneration after anterior cruciate ligament transection (ACLT) in mice. **(A)** HE staining where thickness of hyaline cartilage (HC) and calcified cartilage (CC) are marked by double-headed arrows. Scale bars, 100 μm. **(B)** Safranin O staining is marked with solid arrows and indicate proteoglycan loss and cartilage impairment at 30 and 60 days postoperation. Scale bar, 400 μm (top panels); 200 μm (bottom panels). **(C, D)** HC and CC thicknesses change in different groups and time points. **(E)** Histologic OA scoring of articular cartilage at different time points after surgery. Vehicle = ACLT surgery treated with vehicle. ART = ACLT surgery treated with artesunate. n = 10 per group. Numerical data are presented as means ± SD; **p < 0.01 compared to the sham group, ^##^ *p* < 0.05 compared to the vehicle group.

**Figure 2:**
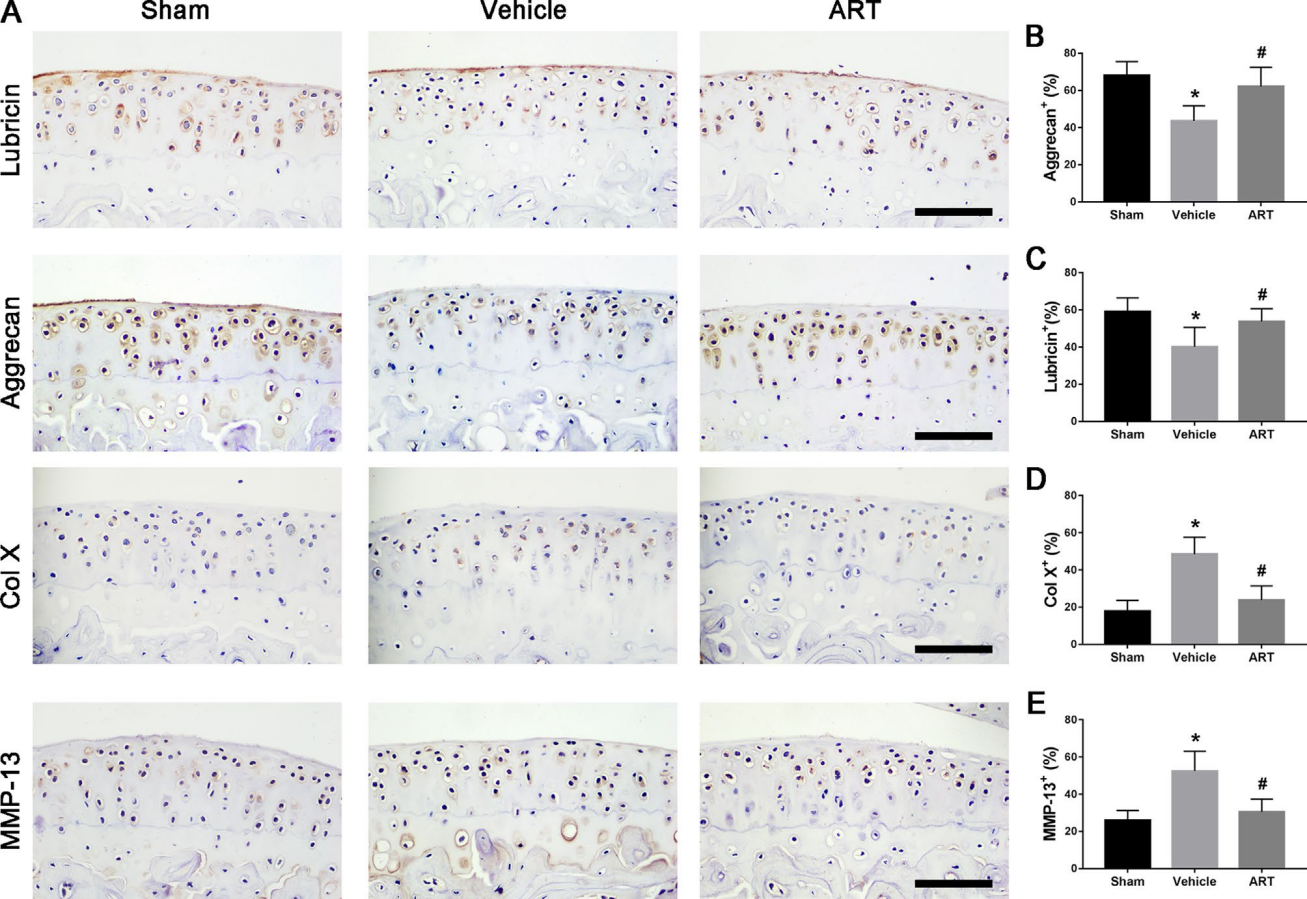
Artesunate (ART) normalizes the number of lubricin, aggrecan, matrix metalloproteinase-13 (MMP-13), and collagen X (COL X) in articular cartilage at 30 days after ACLT surgery. **(A–E)** Immunostaining and quantitative analysis of lubricin, aggrecan, MMP-13, and COL X. Scale bar, 100 μm. n = 10 per group. Means ± SD; *p < 0.05 compared to the sham group, ^#^ *p* < 0.05 compared to the vehicle group.

In a formal experiment, HE staining indicated that ART inhibited the advancement of CC, as exhibited by the upward moving tidemark in ACLT mice at postoperative day 60 (**Figure 1A, C, D**). Safranin O staining showed that loss of proteoglycan was significantly attenuated in the ART group relative to the vehicle group at postoperative day 30 and day 60, which was comparable to that of the sham controls (**Figure 1B**). Additionally, histologic scoring of OA was lowered in ART group compared with vehicle group, and no significant difference was noted in the ART group versus the sham group (**Figure 1E**). Additionally, the results from immunostaining showed that ART normalized the expression of lubricin and aggrecan in articular cartilage compared with the vehicle group, in which these molecule staining was absent or minimal (**Figure 2A–C**). Conversely, abnormal expression of COL X and MMP-13 was observed in the vehicle group relative to the sham group, which was subsided by ART treatment (**Figure 2A, D, E**).

### ART Reestablishes Coupled Subchondral Bone Remodeling in Subchondral Bone

**Figure 3:**
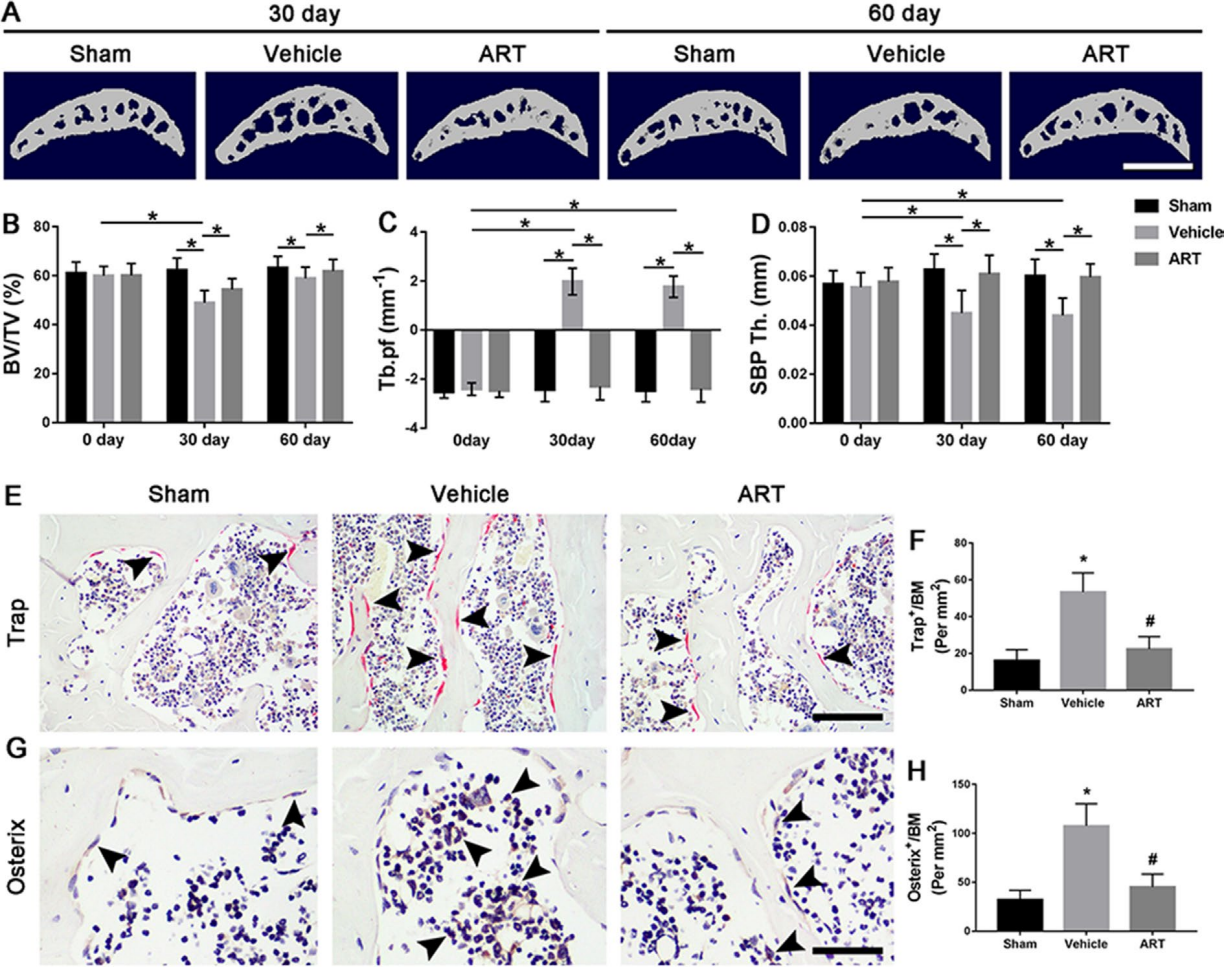
Artesunate (ART) blocks abnormal subchondral bone remodeling after ACLT in mice. **(A)** 3D micro-CT images of sagittal views of subchondral bone medial compartment at 30 and 60 days after sham operation or ACLT surgery. Scale bar, 500 μm. **(B–D)** Quantitative micro-CT analysis of tibial subchondral bone of bone volume fraction (BV/TV), trabecular pattern factor (Tb.pf), and subchondral bone plate thickness (SBP Th). **(E, F)** Immunostaining and quantitative analysis of tartrate-resistant acid phosphatase (TRAP) at 14 days after surgery. Scale bar, 100 μm. **(G, H)**. Immunohistochemical staining and quantification of osterix^+^ cells in subchondral bone at 30 days after surgery. Scale bar, 50 μm. n = 10 per group. Means ± SD; *p < 0.05 compared to the sham group or as denoted by bar, ^#^ *p* < 0.05 compared to the vehicle group.

To explore whether the chondroprotective effects of ART are related to its potential effects on subchondral bone remodeling, µCT and staining were employed to analyze the changes in tibial subchondral bone microarchitecture. The results showed that BV/TV was decreased in ACLT mice compared with sham controls, whereas ART returned a BV/TV value similar to the normal levels (**Figure 3A, B**). Additionally, greater Tb.pf and lower SBP thickness in ACLT mice were restored by ART, and there was no statistically significant difference in these parameters between the ART and sham groups (**Figure 3A, C, D**). In addition, higher Trap^+^ osteoclasts in vehicle-treated ACLT mice were decreased by ART with minimal Trap^+^ cells (**Figure 3E, F**). Consistently, a significantly greater number of osterix^+^ cells were located in the bone marrow cavities of vehicle-treated ACLT mice, while these cells were reduced and relocated to the bone surface in the ART-treated ACLT mice (**Figure 3G, H**).

**Figure 4:**
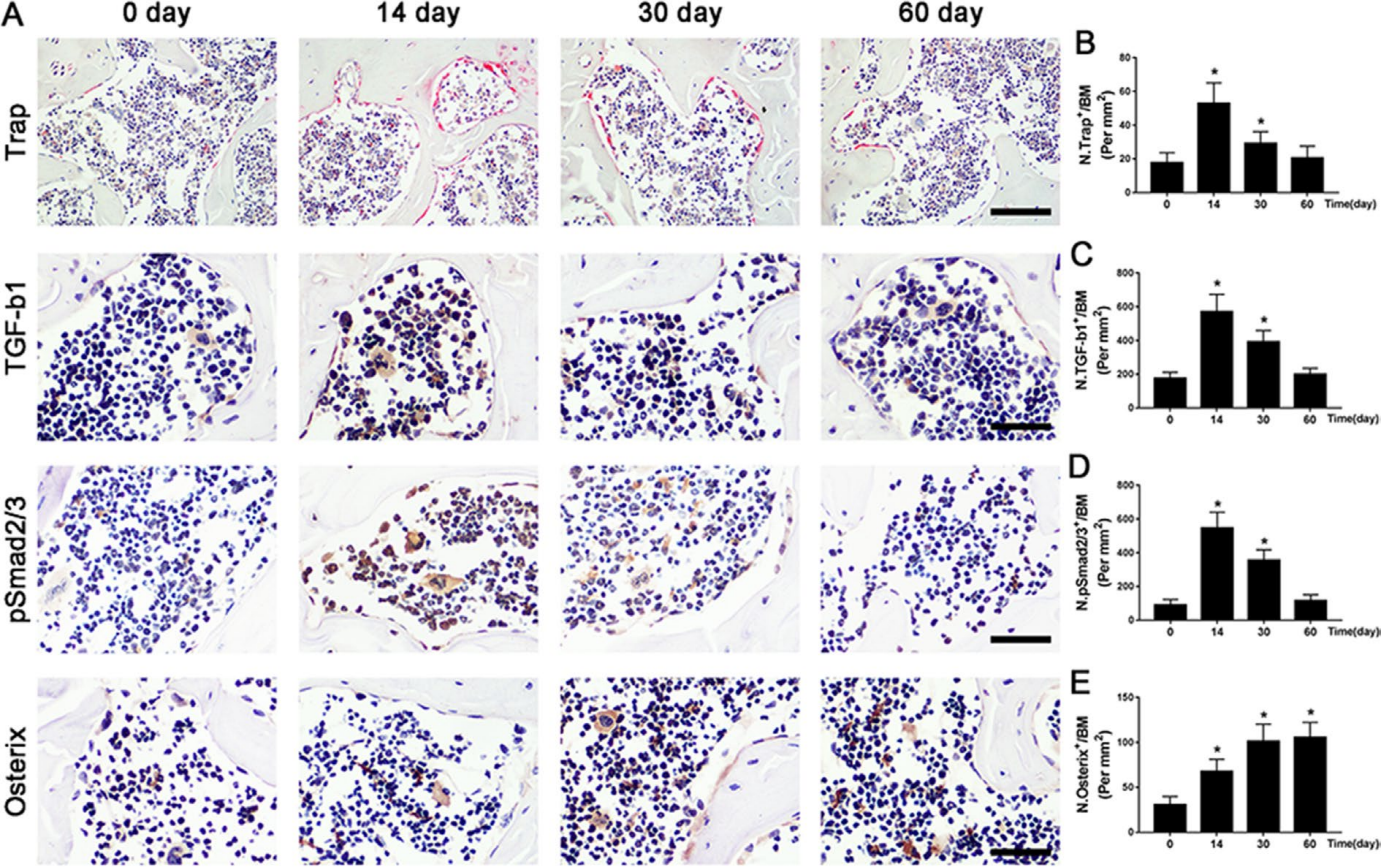
The potential pathogenesis of OA is exhibited in ACLT mice. **(A–D)** Immunostaining and quantitative analysis for TRAP **(A-top, B)**, TGF-β1 **(A-middle, C)**, pSmad2/3^+^ cells **(A-middle, D)**, and osterix^+^ cells** (A-bottom, E)** in sagittal sections of subchondral bone medial compartment at 0, 14, 30, and 60 days after ACLT surgery. Scale bar, 50 μm. n = 10 per group. Means ± SD; *p < 0.05 compared to the sham group, ^#^ *p* < 0.05 compared to the vehicle group.

To further illustrate the potential molecular mechanism of OA, we analyzed ACLT mice models at different time points. Trap^+^ osteoclasts, active TGF-β1, and pSmad2/3^+^ cells were greater at 14 days compared to baseline at day 0 and maintained high levels until 30 days and finally returned to approximate baseline numbers by 60 days (**Figure 4A–D**). Osterix^+^ cells increased in the bone marrow from 14 days to 60 days after surgery (**Figure 4A, E**), indicating that increased osterix^+^ osteoprogenitors likely contributed to *de novo* bone formation.

These results indicated that bone remodeling was elevated in the subchondral bone of ACLT mice, whereas ART significantly attenuated subchondral bone remodeling in early OA.

### ART Suppresses Osteoclastogenesis by Regulating the RANKL-OPG System in Subchondral Bone

**Figure 5:**
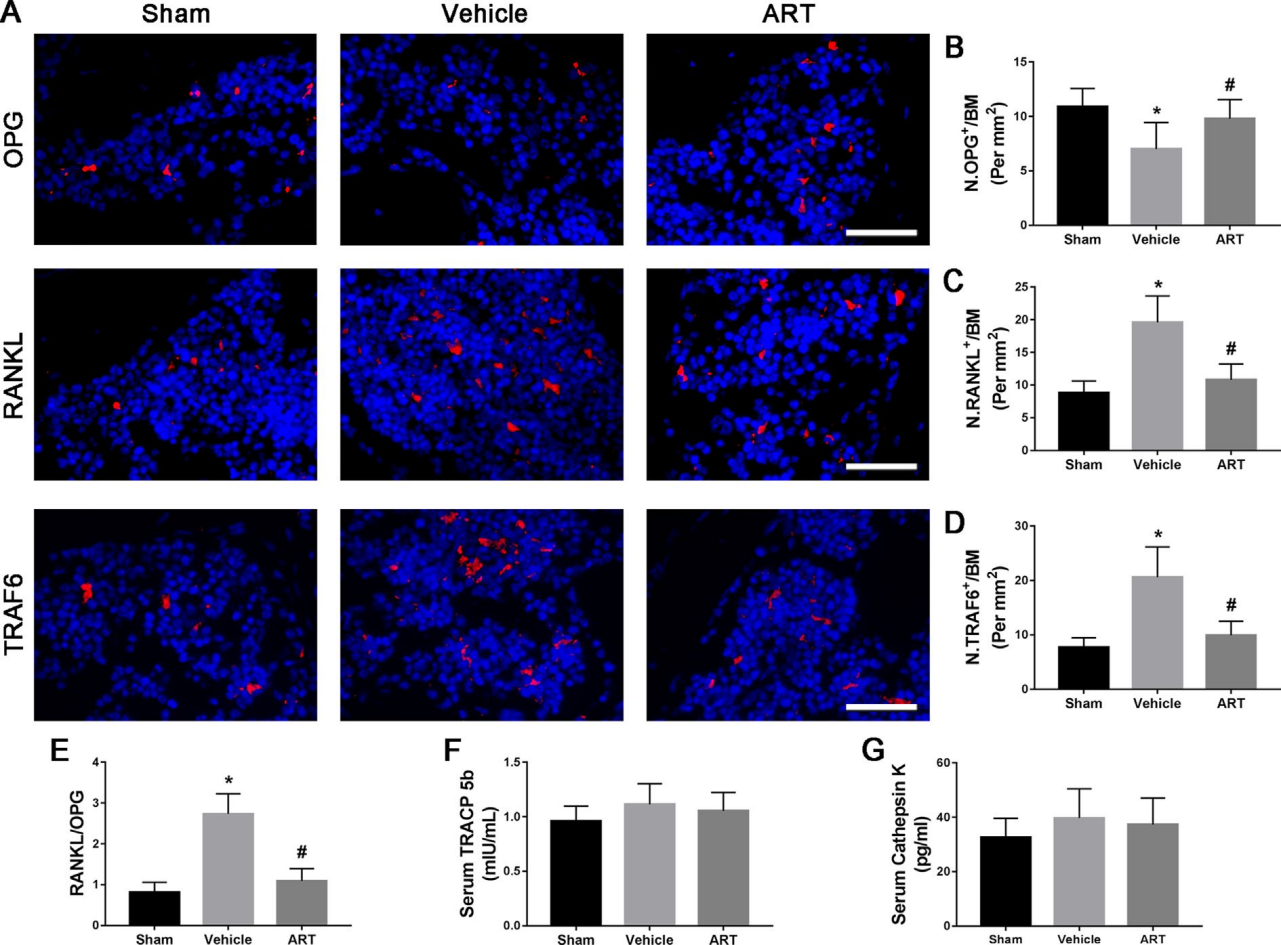
Artesunate (ART) regulates the RANKL-OPG system in the subchondral bone of ACLT mice. **(A–D)** Representative immunofluorescence staining and quantitative analysis for OPG, RANKL, and TRAF6 at 14 days postoperation. Scale bar, 50 μm. **(E)** The ratio of RANKL to OPG in the subchondral bone. **(F, G)** ELISA analysis of TRAP5b and cathepsin K in peripheral blood at 14 days postsurgery. n = 10 per group. Means ± SD; *p < 0.05 compared to the sham group, ^#^ *p* < 0.05 compared to the vehicle group.

To study the potential mechanism of how ART affects the microarchitecture of subchondral bone, immunofluorescence staining and ELISA were performed. The findings showed a significant increase in RANKL and TRAF6 and in RANKL/OPG ratio in subchondral bone marrow of vehicle group at postoperative day 14, whereas ART group had noticeably lowered the expression of RANKL and TRAF6 and the RANKL/OPG ratio, similar to the level of sham group (**Figure 5A, C–E**). Meanwhile, the expression of OPG in the vehicle group was reduced, which was improved in the ART group, whereas no difference was noted in ART versus sham group (**Figure 5A, B**). The ELISA results showed that the levels of TRAP5b and cathepsin K in the peripheral blood of vehicle-treated ACLT mice showed a growing trend, and ART inhibited this effect, although no significant difference was found among the three groups (**Figure 5F, G**). Additionally, the PFQEP domain in the RANK cytoplasmic motifs is responsible for initiating the NF-κB, c-Jun N-terminal kinase, extracellular signal-regulated kinase, and p38 signaling pathways (Liu et al., 2004). Our molecular docking experiment found that ART formed some favorable connections and docked comfortably with RANK (**Supplementary Figure 2A–C**). Specifically, ART bound with Pro-468 by hydrogen bond and Phe-471 by hydrogen bonding and carbon-hydrogen bonding (**Supplementary Figure 2C**). Collectively, these findings indicated that ART suppressed osteoclastogenesis by regulating the RANKL-OPG system in subchondral bone.

### ART Represses TGF-β Release in Subchondral Bone by Suppressing Osteoclastic Formation

**Figure 6:**
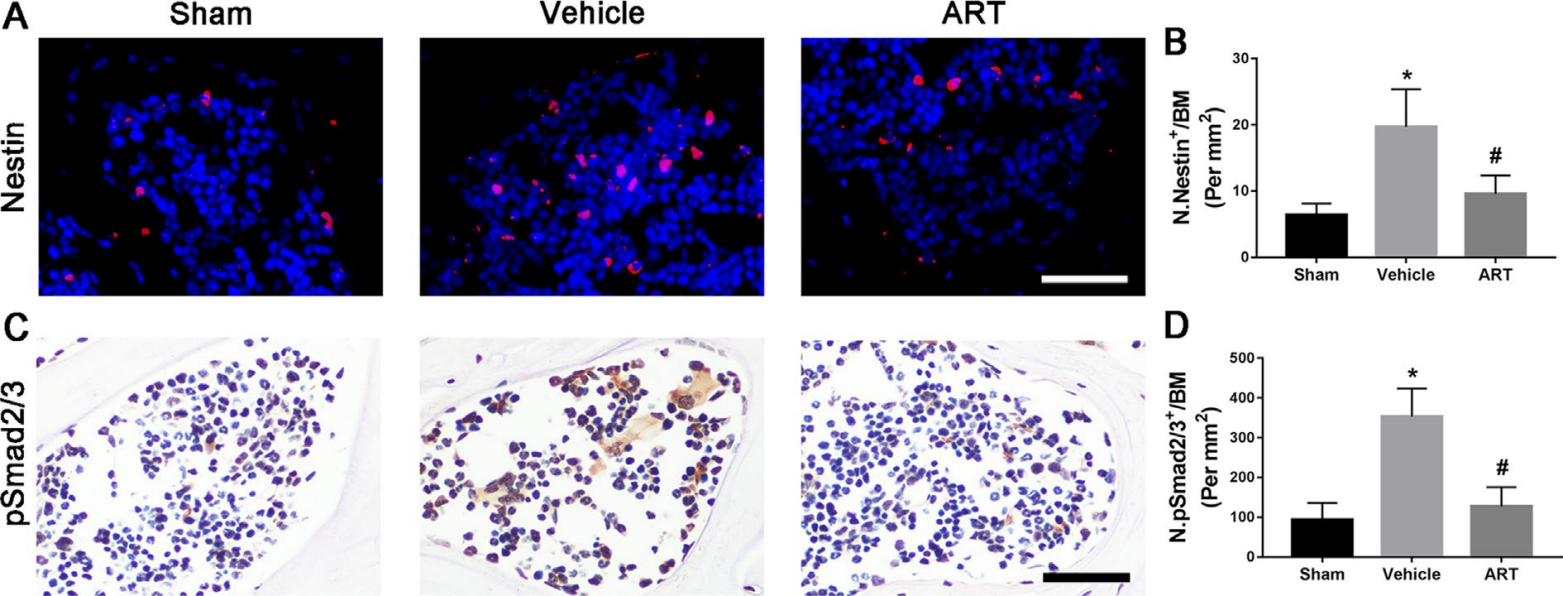
Artesunate (ART) restores coupled bone remodeling in subchondral bone after ACLT in mice. **(A, B)** Immunostaining and quantitative analysis for nestin in sagittal sections of subchondral bone medial compartment at 30 days postoperation. Scale bar, 50 μm. **(C, D)** Immunostaining and quantitative analysis for pSmad2/3^+^ cells in sagittal sections of subchondral bone medial compartment at 30 days postoperation. Scale bar, 50 μm. n = 10 per group. Means ± SD; *p < 0.05 compared to the sham group, ^#^ *p* < 0.05 compared to the vehicle group.

Immunostaining showed that ART significantly reduced the number of nestin^+^ MSCs in subchondral bone post ACLT at postoperative day 30 compared with vehicle, and the difference between the number of nestin^+^ cells in the ART group compared with the sham group was not statistically significant (**Figure 6A, B**). As high concentrations of TGF-β1 induce the recruitment of nestin^+^ MSCs, which further forms the osteoid islets and capillaries in subchondral bone marrow, we investigated whether ART could inhibit Smad2/3 phosphorylation in MSCs. Immunostaining indicated that pSmad2/3^+^ cells in subchondral bone were significantly increased in the vehicle group and attenuated with ART to levels comparable with those in the sham group (**Figure 6C, D**). Furthermore, we performed computational molecular docking using a mice TGF-β receptor I domain model to determine whether ART might bind directly to TGF-β receptor I. A previous study demonstrated that the glycine-serine repeat (GS) domain in TGF-β receptor I is the phosphorylation site functioning as a docking site for signal transduction molecules (Chen et al., 1998). Our results revealed that ART interacted well with TGF-β receptor I (**Supplementary Figure 3A–C**), particularly Ser-287 (**Supplementary Figure 3C**). These observations demonstrated that ART inhibited TGF-β activity in subchondral bone marrow by suppressing abnormal active bone resorption.

### ART Inhibits CD31^hi^Emcn^hi^ Vessel Formation in Subchondral Bone

**Figure 7:**
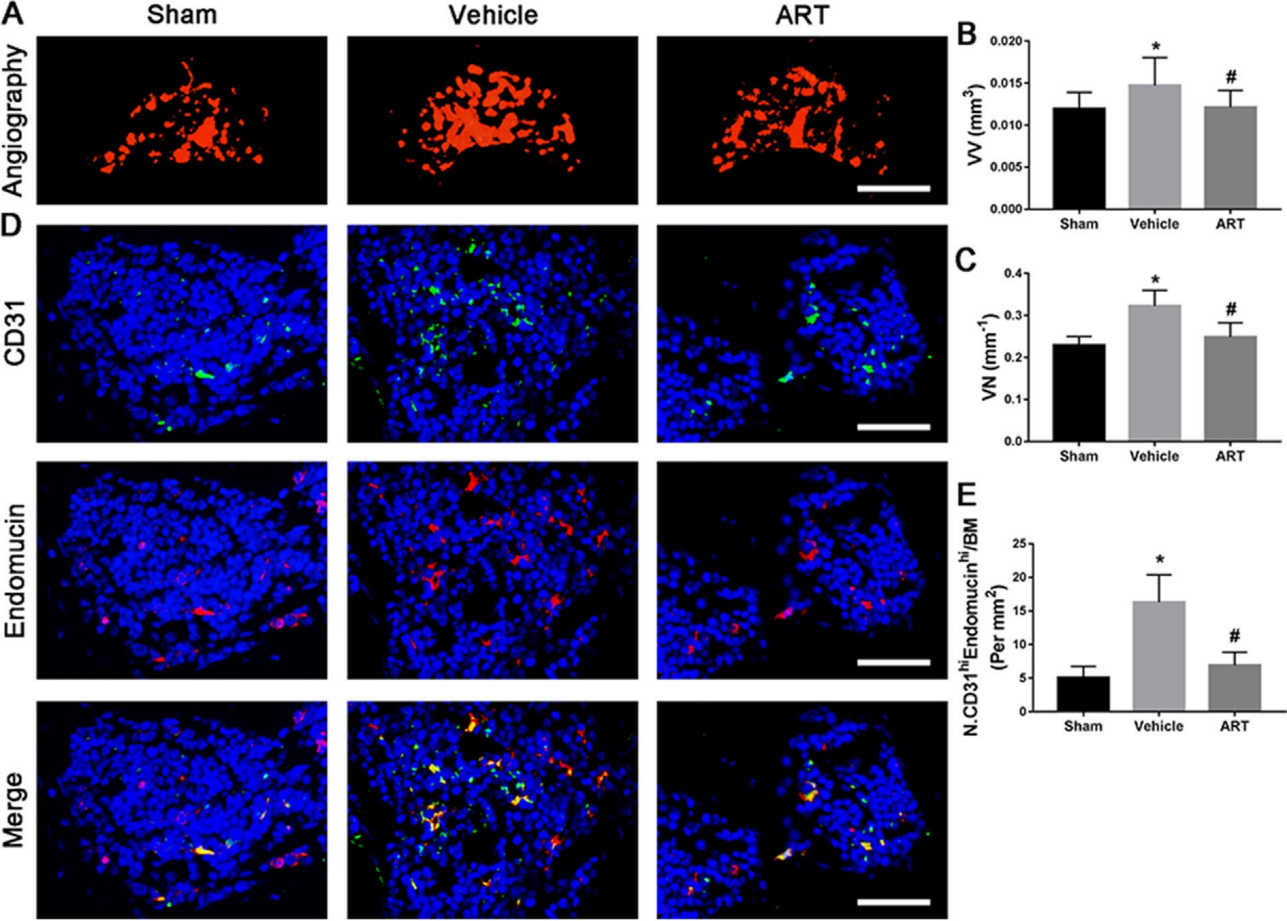
Artesunate (ART) prevents CD31^hi^Emcn^hi^ vessel formation in subchondral bone of ACLT mice. **(A–C)** Three-dimensional CT-based microangiography of medial tibial subchondral bone at 30 days postoperation, with a quantification of vessel volume relative to tissue volume (VV/TV) and vessel number (VN). Scale bar, 500 μm. **(D, E)** Representative immunofluorescence double staining and quantification of CD31^+^ (green) and endomucin^+^ (red) cells at 30 days after surgery. Scale bar, 50 μm. n = 10 per group. Means ± SD; *p < 0.05 compared to the sham group, ^#^ *p* < 0.05 compared to the vehicle group.

The µCT-based angiography was performed to examine the potential effects of ART on aberrant angiogenesis in subchondral bone at postoperative day 30. The results showed less angiogenesis of subchondral bone in the ART group than in the vehicle group. Specifically, ART treatment reduced the number and volume of microvasculature, which was significantly increased in subchondral bone of vehicle group, similar to sham group (**Figure 7A–C**). Immunostaining for CD31 and Emcn was performed to explore the potential mechanism of ART abrogating CD31^hi^Emcn^hi^ vessel formation in subchondral bone. Significant increases in CD31 and Emcn expression were observed in subchondral bone of vehicle-treated mice, whereas the expression levels were attenuated by ART, which were similar to sham controls (**Figure 7D, E**). Additionally, VEGFA and Ang-1 levels were significantly increased in the vehicle group, whereas the ART group had similar VEGFA and Ang-1 levels compared with the sham group (**Figure 7A–D**). Taken together, these results suggested that ART prevented aberrant angiogenesis in subchondral bone as per the experimental protocol followed.

## Discussion

In the present study, we demonstrated the pharmacodynamic effects of ART on articular cartilage degradation and subchondral bone deterioration in OA mice. Specifically, ART reestablished coupled bone remodeling by suppressing active bone resorption and excessive TGF-β release and abrogated CD31^hi^Emcn^hi^ angiogenesis, all of which prevent the recruitment of MSCs for heterotopic osteoid islets in the subchondral bone, ultimately protecting articular cartilage from degeneration and loss and delaying OA progression (**Figure 9**). Furthermore, we clearly illustrated potential cell-signaling mechanisms in the subchondral bone of OA models (**Figures 4** and **9**).

**Figure 8:**
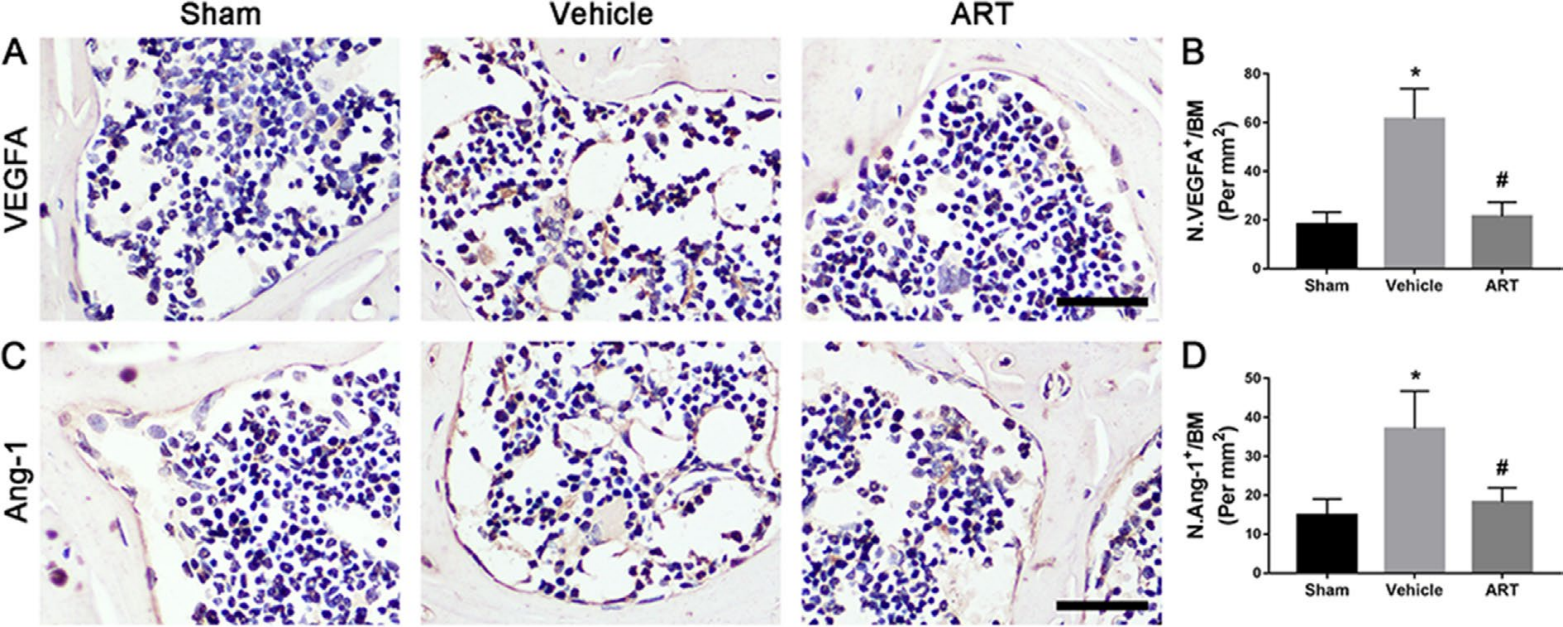
Artesunate (ART) ameliorates excessive expression of vascular endothelial growth factor A (VEGFA) and angiogenin-1 (Ang-1) in subchondral bone of ACLT mice. **(A–D)** Immunostaining and quantitative analysis for VEGFA and Ang-1 in sagittal sections of subchondral bone medial compartment at 30 days postoperation. Scale bar, 50 μm. n = 10 per group. Means ± SD; *p < 0.05 compared to the sham group, ^#^ *p* < 0.05 compared to the vehicle group.

**Figure 9:**
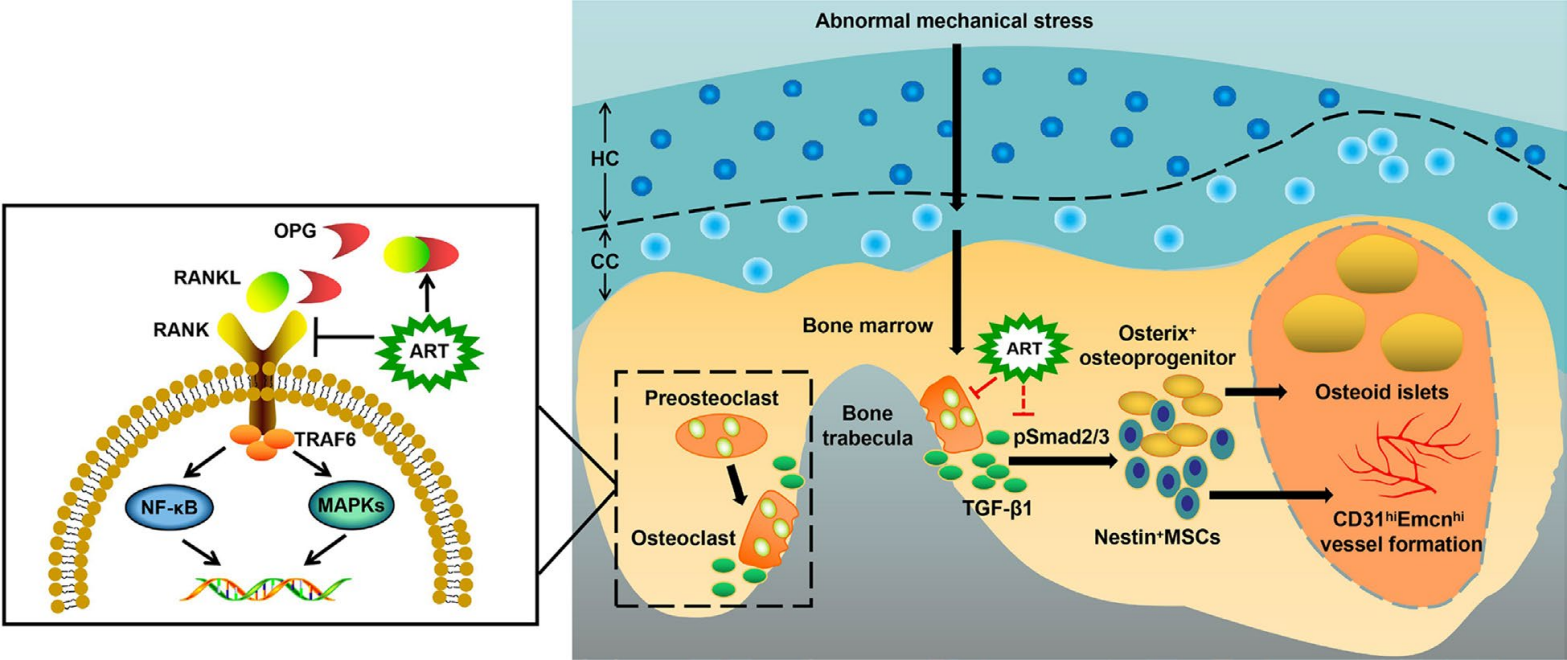
Molecular mechanism of artesunate (ART) on delaying the progression of osteoarthritis (OA). The process of preosteoclast differentiation into osteoclasts is regulated by RANKL and OPG. The binding of RANKL to its receptor RANK on the surface of preosteoclasts induces the activation of TRAF6, stimulating a cascade of events that lead to the differentiation of osteoclasts, while the binding of RANKL to decoy receptor OPG inhibits the reaction above. Abnormal mechanical stress results in hyperactive bone resorption to release excessive TGF-β1, which causes an increase in the number of MSCs and osteoprogenitors in the bone marrow, leading to aberrant osteoid islets and CD31^hi^Emcn^hi^ vessel formation for OA progression. ART inhibits osteoclastic bone resorption by regulating the RANKL-OPG system, restores coupled bone remodeling through suppressing TGF-β1 release, which prevents a cascade of events that accelerate OA progression.

Currently, it is not fully understood that the sequential relationship of changes between articular cartilage and subchondral bone in early OA. Here, we found that subchondral bone underwent abnormal bone remodeling in response to the abnormal mechanical loading, which was induced by ACLT. Only 14 days after surgery, Trap^+^ osteoclasts exhibited a dramatic rise, and hyperactive bone resorption enlarged bone marrow cavities by 30 days. Osterix^+^ osteoblasts also showed a gradual increase at the same time and were primarily located in the bone marrow rather than the bone surface (**Figure 4**). Therefore, µCT results showed hyperactive bone remodeling at postoperative day 30 (**Figure 3A–D**). However, although the reduced proteoglycan in articular cartilage was also observed at postoperative day 30 (**Figure 1B**), thickness of CC zone had no prominent variation until 60 days after ACLT operation (**Figure 1A**). Combined, these observations not only demonstrated that the abnormal bone remodeling in subchondral bone may precede cartilage degradation, but also further supported that there is a closed interplay between subchondral bone and cartilage.

During OA development, active osteoclasts exert catabolic effects in subchondral bone through secretion of hydrochloric acid and proteases, mainly H^+^ and Cl^-^ and cysteine protease cathepsin K (Ono and Nakashima, 2018), which are crucial for TGF-β release from the bone matrix. Thus, inhibition of osteoclast activity may be an important target for OA therapy. Evidence from OA human study suggests a key role of RANKL-OPG system and alteration of their ratio in OA pathology (Tat et al., 2009). When RANKL combines with RANK on preosteoclasts, the adaptor molecules TNF receptor associated factors (TRAFs) are recruited and then activate a series of downstream signaling transcription factors, including NF-κB and MAPKs (p38, JNK, and ERK1/2), to initiate osteoclastogenesis and to coordinate osteoclast functions (Nakashima and Takayanagi, 2011). OPG, a decoy receptor, exerts an essential physiological role in inhibiting osteoclast differentiation and binds RANKL to limit the activation of osteoclast formation (Pantsulaia et al., 2010). In concordance with serum observations in an osteoporosis model (Zeng et al., 2017), our results indicated that ART treatment not only restored the expression of OPG, but also reduced the expression of RANKL and decreased the RANKL/OPG ratio to a level comparable to that in sham group, thereby blocking osteoclastogenesis and bone resorptive function. Moreover, the results from the molecular model revealed that the connection of RANK with downstream molecules may be broken by ART. Among the recruited TRAFs, TRAF6 plays a principal role in osteoclast activation as the main adaptor molecule inside the cell that links to RANK (Darnay et al., 2007). We found that the inhibition of interaction between RANKL and RANK reduced the recruitment of TRAF6 in ART-treated ACLT mice. Moreover, a more recent investigation demonstrated that ART directly repressed TRAF6 expression to inhibit autophagic activation of macrophage, which is a precursor to osteoclast (Kuang et al., 2018). These evidence indicate that ART blocks osteoclast formation and function by inhibiting TRAF6 expression directly and indirectly. Interestingly, several studies have confirmed that ART suppresses osteoclast formation by blocking the NF-κB and the PLCγ1-Ca^2+^-NFATc1 signaling pathways *in vitro* (Zeng et al., 2017; Wei et al., 2018), indicating that ART has powerful potential to inhibit osteoclastogenesis by repressing multiple signaling pathways. Additionally, we found no difference in the levels of TRAP5b and cathepsin K in peripheral blood among the three groups at postoperative day 14, which was different from the osteoporosis model. We speculated that local bone resorption in subchondral bone of OA mice may not be sufficient to elicit changes in the value of TRAP5b and cathepsin K in peripheral blood.

Normal bone remodeling in subchondral bone plays a key role in adapting mechanical stress changes, implicating bone matrix dissolution, and regeneration regulated by osteoclasts and osteoblasts (Castañeda et al., 2012). However, aberrant mechanical loading during the onset of OA results in hyperactive bone resorption, which then releases high concentrations of active TGF-β from the bone matrix (Fang et al., 2016). Subsequently, TGF-β induce differentiation of MSCs into the osterix^+^ osteoprogenitors in bone marrow cavities (Zhen et al., 2013). Ultimately, these clustered osteoblast precursors forms osteoid islets in the subchondral bone marrow, visualized as bone marrow lesions in the magnetic resonance imaging (MRI) and regarded as a prognostic factor of OA (Suri and Walsh, 2012). Our findings revealed that ART-mediated inhibition of abnormal bone resorption resulted in the reduction of TGF-β release, which further decreased nestin^+^ MSC numbers and relocated osterix^+^ osteoprogenitors from the bone marrow to the bone surface, thus restoring coupled bone remodeling. Although ART has been proved to interrupt TGF-β signaling in pulmonary fibrosis models (Li et al., 2014; Wang et al., 2015), the mechanism of whether the ART directly inhibit phosphorylation of Smad2/3 (pSmad2/3) in MSCs in ACLT mice needs be further elucidated in the following study. However, a molecular model revealed that the impairment of TGF-β receptor I phosphorylation may be induced by ART, which provided evidence for subsequent studies.

Aberrant microangiogenesis in subchondral bone is regarded as a pathological feature of OA (Wang et al., 2017). The data obtained from micro-CT showed that the volume and number of microvessels were increased in ACLT mice. In parallel with the results of microangiography, CD31^hi^Emcn^hi^ vessels, a specific vessel subtype coupling osteogenesis (Kusumbe et al., 2014), significantly increased in the subchondral bone marrow. These results further proved the role of aberrant microangiogenesis in subchondral bone during the onset of OA. A recent study found ART-mediated inhibition of neovascularization by inducing ROS-dependent apoptosis in vascular endothelial cells (Cheng et al., 2013). Similarly, our results showed that ART abrogated microvascular invasion, decreased both the volume and number of capillaries in subchondral bone. VEGF and Ang-1 regulate vessel formation at different levels, with VEGF inducing vessel sprouting and growth and Ang-1 mediating vessel remodeling and maturation (Thurston et al., 1999). Specifically, these factors were detected in bone remodeling sites (Horner et al., 2001). We found that ART normalized high levels of VEGFA and Ang-1 in ACLT mice. In addition, previous evidence indicated that TGF-β signaling in endothelial progenitor cells induces angiogenesis (Cunha and Pietras, 2011), but abrogation of TGF-β1 activity can reduce angiogenesis in subchondral bone in OA mice (Zhen et al., 2013). Combined, these data revealed that the profound effect of ART on the inhibition of CD31^hi^Emcn^hi^ vessel formation in subchondral bone was due to the inhibition of increased VEGFA and Ang-1 levels and activated TGF-β signaling.

Artemisinin has been used in Chinese herbal medicine for more than 2,000 years (Kremsner and Krishna, 2004). The small molecule ART, a derivative of artemisinin, has been used to treat various kinds of diseases ranging from malaria to rheumatoid arthritis (Li et al., 2013; Jiang et al., 2018). Our findings further broaden the potential application of ART. In this study, for the first time, it was shown that ART attenuated subchondral bone deterioration, including suppressing dramatic bone resorption, inhibiting heterotopic bone formation through interrupting TGF-β signaling and abrogating aberrant blood vessel formation in the subchondral bone in early-stage OA. Specifically, articular cartilage degeneration was alleviated, indicating that maintaining stabilization of subchondral bone microarchitecture in early OA could be seen as an effective therapeutic approach. Additionally, several limitations were present in this study. Compared with oral administration, intraperitoneal injection is not a considerably more convenient route, especially for those who need to repeatedly administer medications. Although the molecular model indicates the interaction between ART and RANK as well as TGF-β receptor I, this effect needs to be further demonstrated by transgenic and knock-out/in models.

## Data Availability Statement

The datasets for this manuscript are not publicly available because This study will be followed up. Requests to access the datasets should be directed to xjbone@sina.com.

## Author Contributions

YL, WM, and LC designed the research and wrote the paper. YL, WM, BX, TW, and SW performed the experiments. JR, HM, HG, and AA analyzed the data and edited the paper. HM, YL, KZ, and LC contributed the materials and reagents. YL, WM, and LC revised the paper and guided the research.

## Funding

This study was supported by grants from Joint Funds of the National Natural Science Foundation of China (No. U1503221 and No. 81860746), High-end Talent Team Construction Foundation (No. 108-10000318), and Xinjiang Natural Science Foundation (No. 2018D01C187).

## Conflict of Interest Statement

The authors declare that the research was conducted in the absence of any commercial or financial relationships that could be construed as a potential conflict of interest.

## Supplementary Material

Supplementary Figure 1 In a preliminary experiment, artesunate (**ART**) ameliorates articular cartilage degeneration after anterior cruciate ligament transection (**ACLT**) in mice. **(A, C)** Changes in the thickness of calcified cartilage (**CC**) and hyaline cartilage (**HC**) in HE staining. Scale bars, 100 μm. **(B, D)** Safranin O staining and histologic OA scoring of articular cartilage in different concentration groups at 60 days postoperation. Scale bar, 400 μm (top panels), 200 μm (bottom panels). **(E)** The liver coefficient in different concentration groups 60 days after surgery.

Supplementary Figure 2 Molecular modeling study of the binding of artesunate (ART) binding to RANK. **(A)** Structure of ART. **(B)** Structure of RANK. **(C)** Three-dimensional and two-dimensional modeling of ART binding within the domain of RANK.

Supplementary Figure 3 Molecular modeling study of the binding of artesunate (ART) binding to TGF-b receptor I. **(A)** Structure of ART. **(B)** Structure of TGF-b receptor I. **(C)** Three-dimensional and two-dimensional modeling of ART binding within the domain of TGF-b receptor I.
