# Supplementary figures and images for: Artesunate, an Anti-Malaria Agent, Attenuates Experimental Osteoarthritis by Inhibiting Bone Resorption and CD31hiEmcnhi Vessel Formation in Subchondral Bone
Source: Front Pharmacol. 2019 Jun 14;10:685. doi: 10.3389/fphar.2019.00685 (PMC6587439; doi:10.3389/fphar.2019.00685)

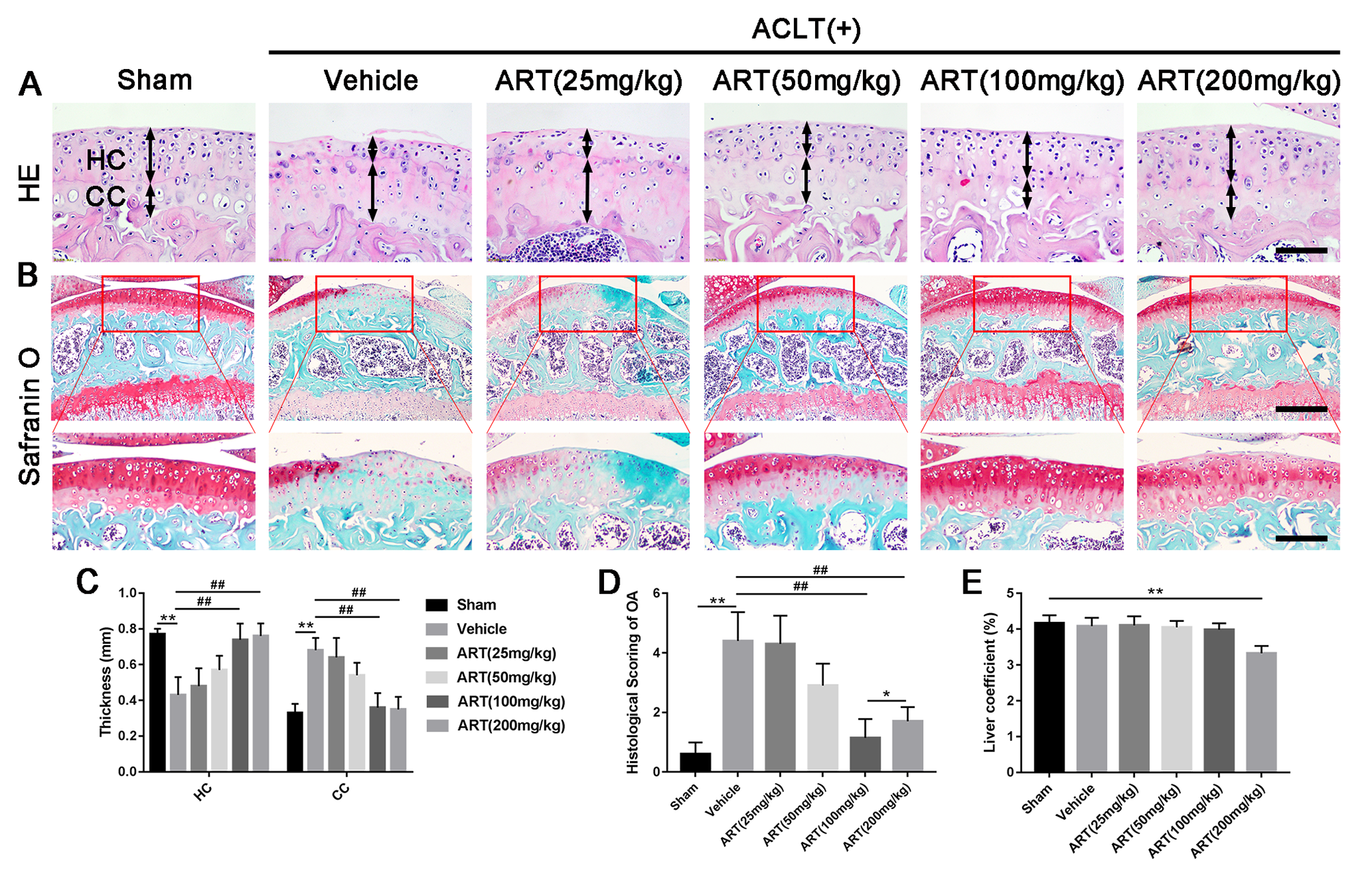

Supplement: Supplementary Figure 1 — In a preliminary experiment, artesunate (ART) ameliorates articular cartilage degeneration after anterior cruciate ligament transection (ACLT) in mice. (A, C) Changes in the thickness of calcified cartilage (CC) and hyaline cartilage (HC) in HE staining. Scale bars, 100 μm. (B, D) Safranin O staining and histologic OA scoring of articular cartilage in different concentration groups at 60 days postoperation. Scale bar, 400 μm (top panels), 200 μm (bottom panels). (E) The liver coefficient in different concentration groups 60 days after surgery. [file Image_1.tif]

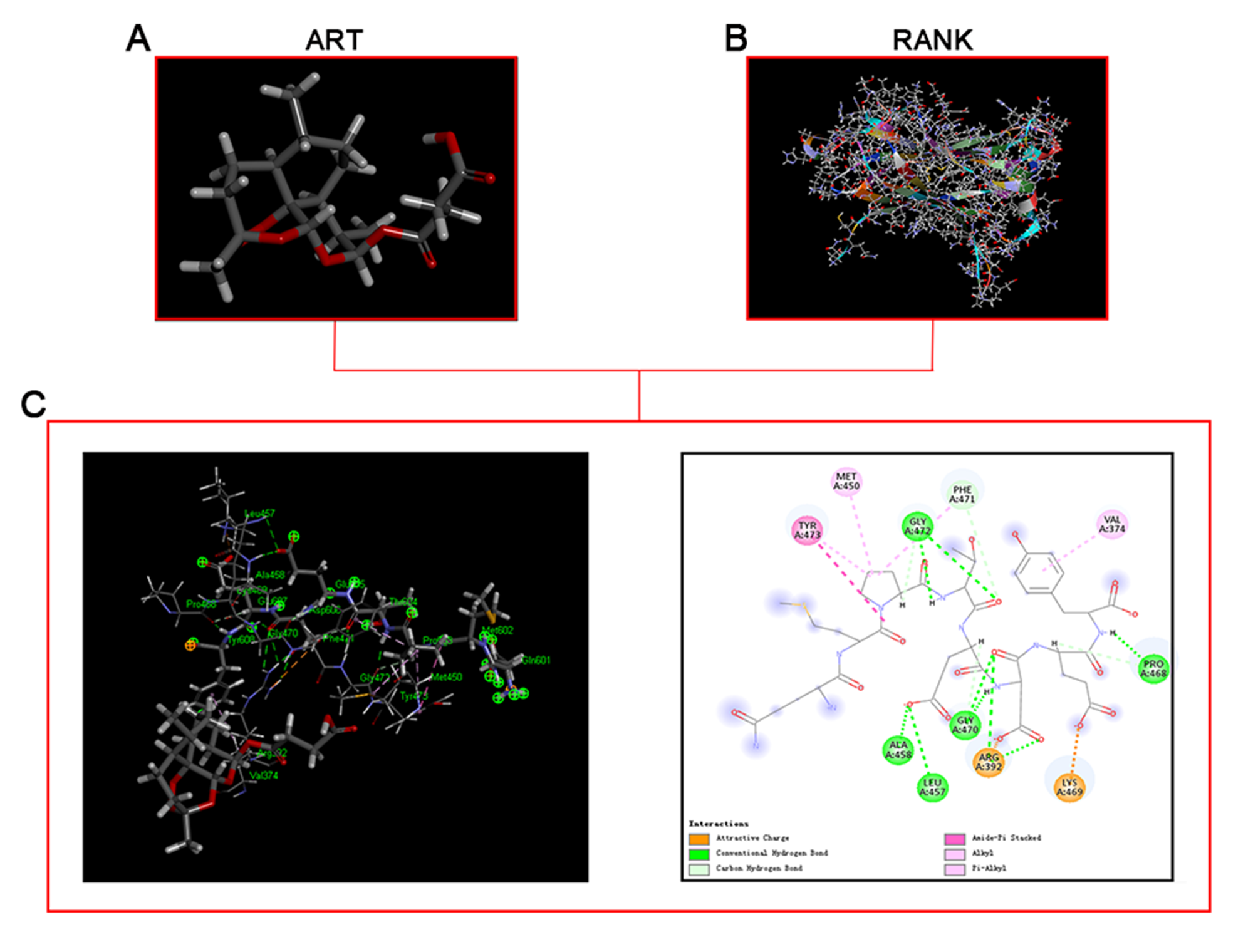

Supplement: Supplementary Figure 2 — Molecular modeling study of the binding of artesunate (ART) binding to RANK. (A) Structure of ART. (B) Structure of RANK. (C) Three-dimensional and two-dimensional modeling of ART binding within the domain of RANK. [file Image_2.tif]

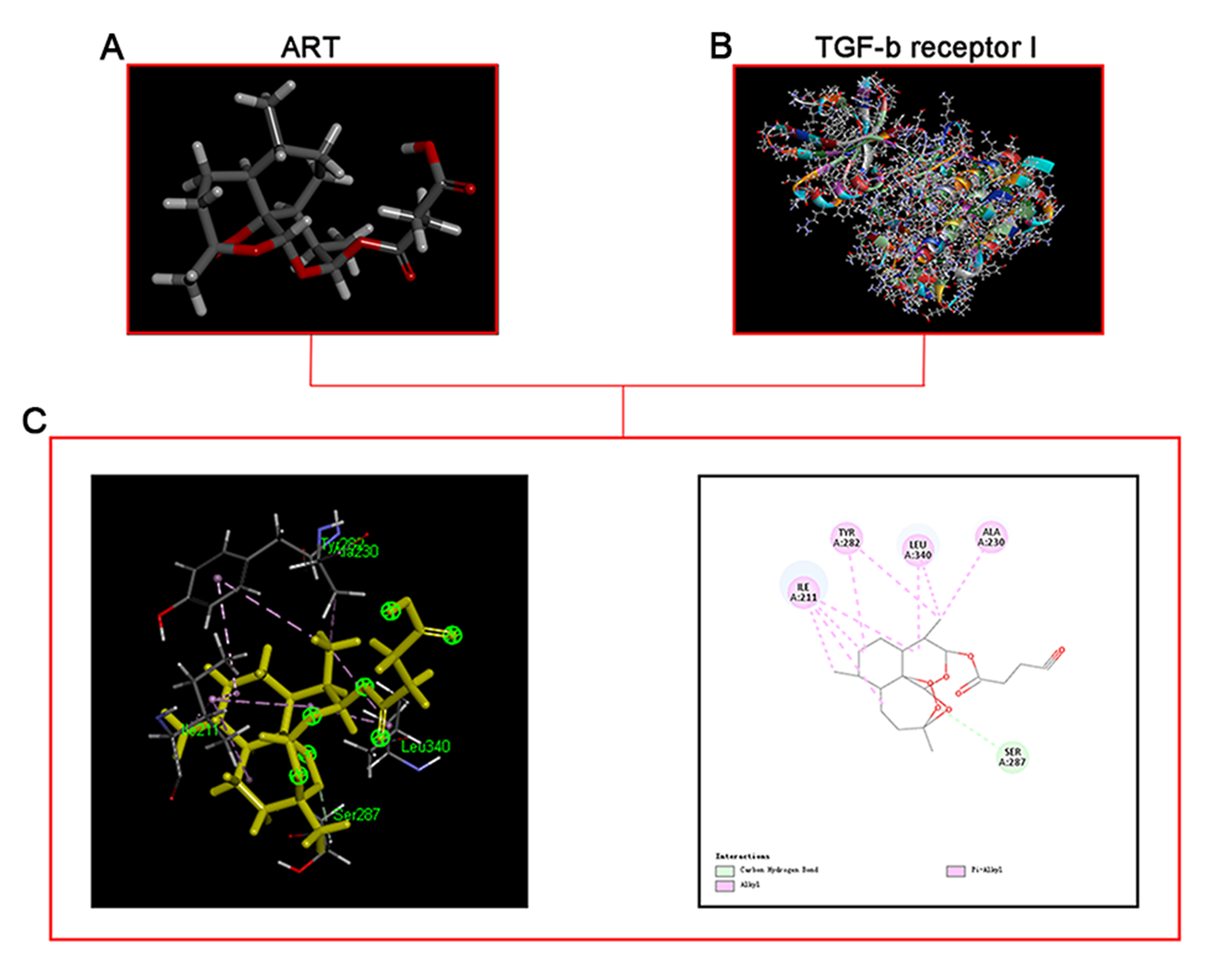

Supplement: Supplementary Figure 3 — Molecular modeling study of the binding of artesunate (ART) binding to TGF-b receptor I. (A) Structure of ART. (B) Structure of TGF-b receptor I. (C) Three-dimensional and two-dimensional modeling of ART binding within the domain of TGF-b receptor I. [file Image_3.tif]
